# Durable reduction of *Clostridioides difficile* infection recurrence and microbiome restoration after treatment with RBX2660: results from an open-label phase 2 clinical trial

**DOI:** 10.1186/s12879-022-07256-y

**Published:** 2022-03-12

**Authors:** Robert Orenstein, Erik R. Dubberke, Sahil Khanna, Christine H. Lee, David Yoho, Stuart Johnson, Gail Hecht, Herbert L. DuPont, Dale N. Gerding, Ken F. Blount, Sarah Mische, Adam Harvey

**Affiliations:** 1 Division of Infectious Diseases, Mayo Clinic in Arizona, 5777 e Mayo Blvd, Phoenix, AZ 85054 USA; 2 Department of Medicine, Washington University School of Medicine, St. Louis, MO USA; 3 Division of Gastroenterology and Hepatology, Mayo Clinic, Rochester, MN USA; 4 Hamilton Regional Laboratory Medicine Program, Department of Pathology and Molecular Medicine, McMaster University, Hamilton, ON Canada; 5 Infectious Diseases, Mid-Atlantic Permanente Medical Group, Springfield, VA USA; 6 Infectious Disease, Loyola University Medical Center, Chicago, IL USA; 7 Edward Hines Jr. VA Hospital, Hines, IL USA; 8 Division of Gastroenterology, Hepatology and Nutrition, Loyola University Medical Center, Maywood, IL USA; 9 University of Texas Health Science Center and Kelsey Research Foundation, Houston, TX USA; 10 Rebiotix Inc., a Ferring Company, Roseville, MN USA; 11 Vancouver Island Health Authority, Victoria, Canada; 12 Department of Pathology and Laboratory Medicine, University of British Columbia, Vancouver, BC Canada

**Keywords:** *Clostridioides difficile*, Recurrence, Microbiota-based therapy, Clinical trial, Durability, Response

## Abstract

**Background:**

Effective treatment options for recurrent *Clostridioides difficile* infection (rCDI) are limited, with high recurrence rates associated with the current standard of care. Herein we report results from an open-label Phase 2 trial to evaluate the safety, efficacy, and durability of RBX2660—a standardized microbiota-based investigational live biotherapeutic—and a closely-matched historical control cohort.

**Methods:**

This prospective, multicenter, open-label Phase 2 study enrolled patients who had experienced either ≥ 2 recurrences of CDI, treated by standard-of-care antibiotic therapy, after a primary CDI episode, or ≥ 2 episodes of severe CDI requiring hospitalization. Participants received up to 2 doses of RBX2660 rectally administered with doses 7 days apart. Treatment success was defined as the absence of CDI diarrhea without the need for retreatment for 8 weeks after completing study treatment. A historical control group with matched inclusion and exclusion criteria was identified from a retrospective chart review of participants treated with standard-of-care antibiotics for recurrent CDI who matched key criteria for the study. The primary objective was to compare treatment success of RBX2660 to the historical control group. A key secondary outcome was the safety profile of RBX2660, including adverse events and CDI occurrence through 24 months after treatment. In addition, fecal samples from RBX2660-treated participants were sequenced to evaluate microbiome composition and functional changes from before to after treatment.

**Results:**

In this Phase 2 open-label clinical trial, RBX2660 demonstrated a 78.9% (112/142) treatment success rate compared to a 30.7% (23/75) for the historical control group (p < 0.0001; Chi-square test). Post-hoc analysis indicated that 91% (88/97) of evaluable RBX2660 responders remained CDI occurrence-free to 24 months after treatment demonstrating durability. RBX2660 was well-tolerated with mostly mild to moderate adverse events. The composition and diversity of RBX2660 responders’ fecal microbiome significantly changed from before to after treatment to become more similar to RBX2660, and these changes were durable to 24 months after treatment.

**Conclusions:**

In this Phase 2 trial, RBX2660 was safe and effective for reducing rCDI recurrence as compared to a historical control group. Microbiome changes are consistent with restorative changes implicated in resisting *C. difficile* recurrence.

*Clinical Trials Registration* NCT02589847 (10/28/2015)

**Supplementary Information:**

The online version contains supplementary material available at 10.1186/s12879-022-07256-y.

## Background

Recurrent *Clostridioides difficile* infections (rCDIs) are associated with significant morbidity, mortality, decreased quality-of-life, and substantial costs [1]. The current standards of care are antibiotics, with consideration of fecal microbiota transplant (FMT) after multiple recurrences [2]. Using microbial consortia to restore the composition and diversity of patients’ intestinal microbiota is a promising strategy for preventing rCDI [3], with FMT gaining traction, despite a lack of standardization of process, product, or procedure [4]. Several investigational microbiota-based therapeutics are in clinical development [5, 6], including RBX2660—a standardized, stabilized, microbiota-based investigational live biotherapeutic derived from vigorously screened healthy human fecal donations. In previous studies, RBX2660 reduced CDI recurrence and normalized microbiota composition [3, 6]. Herein we report final efficacy and safety results from a Phase 2 open-label study of RBX2660, including fecal microbiome changes from before to after treatment. RBX2660 reduced CDI recurrence comparably to previous trials, with a similar safety profile and shifted participants’ microbiome to a composition more associated with resisting *C. difficile* colonization, and these clinical and microbiome outcomes were sustained to 24 months after treatment.

## Methods

This prospective Phase 2 open-label study was conducted at 29 medical centers in the United States and Canada under a US Food and Drug Administration Investigational New Drug application and a Health Canada Clinical Trial Application (CTA) (NCT02589847 10/28/2015). The protocol included an active RBX2660 treatment group at all investigative sites, as well as a chart review historical control at four investigative sites. The institutional review board/research ethics board (IRB/REB) at each participating center approved the study protocol and provided a waiver of informed consent for the chart reviews at the four historical control sites. All RBX2660-treated participants provided written informed consent. An independent medical monitor provided safety oversight.

### Study population

The study population included participants ≥ 18 years old with a diagnosis of rCDI and either (a) ≥ 2 documented recurrences of CDI after a primary episode and had completed at least two rounds of standard-of-care oral antibiotic therapy, or (b) ≥ 2 documented episodes of severe CDI resulting in hospitalization. Participants were required to have had a positive stool test for *C. difficile* or its toxins within 60 days prior to enrollment and to already be taking or starting antibiotics for control of rCDI symptoms at the time of enrollment. The choice of *C. difficile* diagnostic test for entry qualification was per the standard of care at each investigative site. Because participants were already taking antibiotics for the most recent CDI episode at the time of enrollment, the course of antibiotics prescribed was chosen by the investigator prior to enrollment and not protocol-specified. After enrollment, participants were required to have rCDI symptoms controlled by antibiotic before receiving study treatment. Major exclusion criteria included: a history of continued CDI diarrhea despite antibiotic treatment for rCDI, planned surgery requiring pre-/perioperative antibiotics, non-CDI related diarrhea, a compromised immune system, a diagnosis of inflammatory bowel disease, or pregnancy.

Historical control participants were identified through a retrospective chart review of patients treated with antibiotics for rCDI who matched key inclusion/exclusion criteria from the active RBX2660 treatment arm at four study sites that also enrolled for the RBX2660 treatment arm. A search of hospital medical records was performed by designated site staff using the relevant diagnosis codes to identify patients diagnosed with CDI. All patient records on the resulting list were reviewed against inclusion/exclusion criteria by a designated, qualified, and trained chart reviewer at each site, with documentation of the review and reason for exclusion of any records from the final historical control data set. For eligible charts, efficacy data including treatment success and safety data were collected from the date of the diagnosis of the first CDI episode through 6 months after resolution of the last episode or the last encounter date, whichever was later.

### RBX2660 preparation and administration

Each dose of RBX2660 comprises a 150 mL (approximately 5 oz) microbiota suspension in a single-dose ready-to-use enema containing a minimum of 10^7^ live organisms/mL. RBX2660 doses were manufactured from human stool as previously described [6] and in accordance with proprietary process and quality controls agreed upon with the FDA within the IND application and in accordance with FDA live biotherapeutic guidelines. Each dose was manufactured from and is traceable to a single donor and donation that have undergone extensive continuous safety screening. The assigned study treatment was two doses of RBX2660 delivered via enema 7 ± 2 days apart, with the first dose administered between 24 and 48 h after completion of CDI antibiotics. The second dose could be administered sooner at the discretion of the investigator if CDI symptoms returned. The two doses were not required to be from the same donor. Administration of the enema can be accomplished with the patient in the left lateral decubitus position (or knee-chest) in approximately 5 min and participants were encouraged to retain the enema as long as feasible (mean time was 43.4 min).

### Study outcomes

The primary efficacy endpoint was treatment success, defined as the absence of CDI diarrhea without the need for retreatment of CDI, determined at a week 8 office visit. Treatment failure (CDI recurrence) was determined by the site investigator, with a protocol definition of meeting all the following criteria: CDI diarrhea, a positive laboratory diagnosis for *C. difficile* or its toxins in stool, a need for CDI retreatment, and no other cause for CDI symptoms. CDI diarrhea was defined as the passage of ≥ 3 watery stools in ≤ 24 h for ≥ 2 consecutive days. In practice, most site investigators did not rule out alternative causes for illness once diarrhea, laboratory *C. difficile* diagnosis, and a need for CDI retreatment were confirmed. After the 8-week efficacy timepoint, the incidence of CDI occurrences was monitored via phone follow-up at the 3-, 6-, 12-, and 24-month timepoints. Treatment success for participants in the historical control group was defined as the absence of CDI recurrence within 8 weeks of completing antibiotic therapy for the study-qualifying CDI episode.

A key secondary outcome was the safety of RBX2660, including adverse events (AEs) and serious AEs (SAEs). AEs were actively collected using a study diary (with data collected from the start of treatment through 7 days after the last study treatment), through in-office visits during RBX2660 administration and at 8 weeks after treatment, and through follow-up phone calls at weeks 1, 2, 3 and 4, and months 3, 6, and 12 after treatment. An additional follow-up phone call at 24 months assessed for SAEs. The AEs and SAEs were categorized by the site investigator for severity, seriousness, and relatedness to RBX2660, the enema procedure, pre-existing conditions, or CDI. Safety data for the historical controls were gathered but not compared to the RBX2660 arm because the defined follow-up was shorter for the historical control and because historical control safety events were not collected systematically or proactively as was done for the RBX2660 arm.

The primary efficacy endpoint was assessed in the Evaluable Population, defined as all participants who experienced a confirmed treatment failure, or reached the 8-week timepoint in order to be determined a treatment success. Safety results for RBX2660 are reported using the Safety population, defined as all enrolled participants which were treated with RBX2660. The full analysis set (FAS) includes the RBX2660 safety population and all enrolled historical controls (excluding screen failures).

### Data monitoring and statistical analysis

An independent medical monitor reviewed AEs and SAEs and provided medical surveillance as needed. Baseline values for demographic, clinical, and outcome variables (primary and secondary) were tabulated to identify potential confounding variables among all participants enrolled in the study who received RBX2660 treatment and for the historical control cohort. The primary efficacy analysis used Pearson’s Chi-square test to compare the recurrence free rate of the RBX2660 and historical control arms. Subgroup analysis of efficacy analysis by demographic subgroups used Fisher’s exact test.

### Microbiome analysis of RBX2660-treated participants

Participation in the sample collection phase of the trial was optional per consent requirements. Participants who opted in were provided kits to collect whole stool samples at home and ship in a freezer pack via overnight courier to Rebiotix, with a request to collect and ship samples prior to the first treatment visit (“BL,” or baseline) and 7, 30, and 60 days and 6, 12, and 24 months after treatment. Upon receipt at Rebiotix, samples were aliquoted and frozen at – 80 °C with no added stabilizers until analysis. To preclude selection bias, all received samples that met specified time point criteria were included in the analysis, with the exception that samples collected after treatment failure were not included, since those participants received antibiotics and/or conventional FMT which would confound analysis of subsequent timepoints. An aliquot of each RBX2660 batch administered to participants in the evaluable population was also included in the sequencing analysis.

RBX2660 and participant samples were extracted via a PowerFecal kit (Qiagen, Germantown, MD) and sequenced using a shallow-shotgun method (Diversigen, Minneapolis, MN). Sequencing reads were quality filtered (Q score of 25) and processed to operational taxonomic units (OTU) data via a proprietary database and pipeline (Diversigent) that required 97% or greater alignment identity. Samples with fewer than 10,000 sequences were discarded and OTU with less than 0.01% of their unique genome regions covered were discarded. Relative taxonomic abundances and alpha diversity for each sample were determined from OTU data, and non-metric multidimensional scaling analysis (NMDS) was used to map all individual samples onto two-dimensional space with a Bray–Curtis dissimilarity metric [7]. Mean relative abundances at the taxonomic class level (π), with upper and lower confidence limits, were calculated by fitting OTU data to a Dirichlet multinomial function [7], and intergroup statistical hypothesis testing was conducted as indicated per comparison. Effect size (ES) for microbiome comparisons was calculated as a modified Cramer’s criterion ϕ [7, 8], with larger ϕ indicating a larger difference in microbiome taxa distributions.

## Results

### Participants

**Fig. 1:**
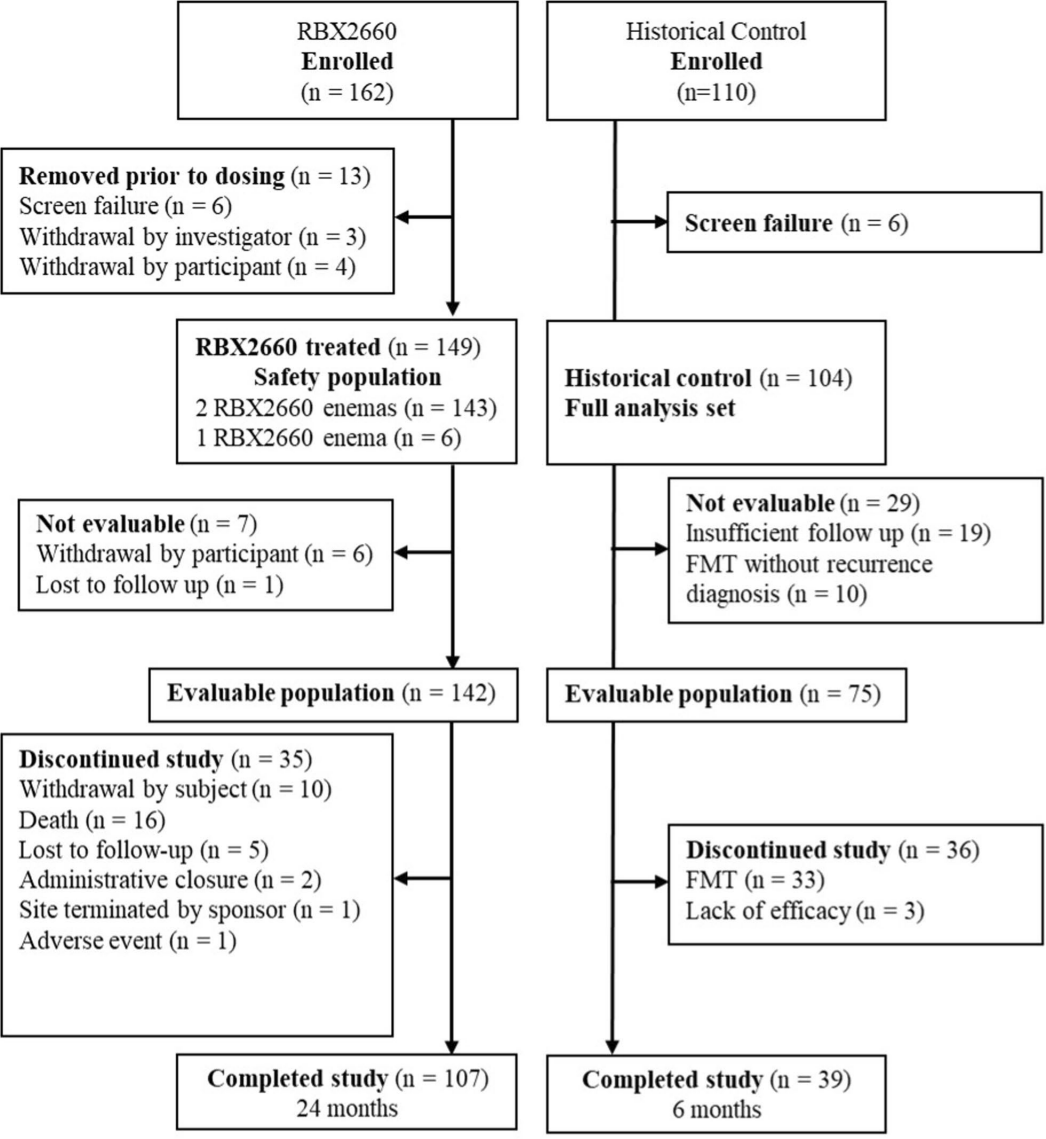
Consort diagram showing participant enrollment, allocation, follow up, and analysis

A total of 162 participants were enrolled for RBX2660 treatment at 29 centers in the United States and Canada, between October 1, 2015 and March 6, 2017. Six were screen failures (enrolled but did not meet full eligibility criteria), and seven withdrew prior to treatment, leaving 149 participants who received RBX2660 (safety population), of whom 143 (95%) received two RBX2660 doses and 6 participants received one RBX2660 dose (Fig. 1). The demographic characteristics of the safety population were consistent with published rCDI studies, including prior studies of RBX2660 (Table 1). The majority of laboratory diagnoses of *C. difficile* for study enrollment were made by polymerase chain reaction (PCR), with fewer by toxin A/B enzyme immunoassay (EIA). Most received oral vancomycin for the enrolling CDI episode. Among the safety population, 142 remained evaluable at the primary efficacy endpoint (evaluable population), and 107 participants completed the study with 24-months of follow-up.

**Table 1:** Participant demographics

Demographics		
	RBX2660 FAS population^1^ (n = 149)	Historical control, FAS population^1^ (n = 104)
Mean age [range]	65.1 [19–103]	67.8 [21–97]
Participants/patients ≥ 65, n (%)	87 (58)	64 (62)
Female, n (%)	95 (64)	71 (68)
Race, white, n (%)	136 (91)	40 (38)^2^
Mean total CDI episodes [range]^3^	3.9 [2–13]	2.9 [2–5]
Mean duration all CDI episodes [range]^3^	23 [1–420]	29 [2–380]
Enrollment diagnostics, n (%)^3^		
PCR	98 (66)	82 (83)
Enzyme Immunoassay (EIA)	35 (23)	8 (8)
Other^4^	16 (11)	9 (9)
Antibiotic at enrolling CDI episode, n (%)^3^		
Vancomycin	120 (81)	62 (63)
Fidaxomicin	8 (5)	5 (5)
Metronidazole	10 (7)	6 (6)
Other^5^	11 (7)	26 (26)
Received two RBX2660 doses, n (%)	143 (96)	NA
Received only one RBX2660 dose, n (%)	6 (4)	NA

^1^Includes all RBX2660-treated participants and the enrolled historical control participants, less six screen failures

^2^37 of the 104 Historical Control participants had race “Not Reported”

^3^Five historical control participants in the FAS (3 participants in evaluable population) had incomplete CDI history records and were not able to be categorized

^4^Category ‘other’ for diagnostics included medical record documentation as antigen, empirically tested, loop-mediated isothermal amplification (LAMP), molecular assay, nucleic acid amplification, toxin, toxin with PCR reflex, not reported, or unknown

^5^Category ‘other’ for Antibiotics included medical record documentation as any combination of vancomycin, fidaxomicin, metronidazole, rifampin, and rifaximin, as well as unknown and not reported

A total of 110 participants were enrolled in the historical control arm at four sites, of which six were screen failures, leaving a full analysis set of 104 participants. Study start dates ranged from March 14, 2010 to November 7, 2016 with a median start date of June 25, 2014. Demographics of the historical control cohort were comparable to the RBX2660 safety population, with the exception of race (Table 1). Most were categorized as non-white, but this included “not reported” since the race information was inconsistently documented in the medical charts reviewed. Among the full analysis set, 29 were excluded from the primary efficacy analysis due to not having sufficient data to adjudicate a primary efficacy outcome, leaving an evaluable population of 75.

### Primary efficacy and sustained CDI recurrence-free rate

At 56 days after treatment, the recurrence-free rate among the RBX2660-treated evaluable population was 78.9%, significantly higher than the historical control arm (30.7%, *p* < 0.0001, Table 2). Thus, the primary efficacy endpoint was met.

**Table 2:** Efficacy outcomes

	RBX2660 (n = 142)	Historical control (n = 75)
Recurrence-free rates at 8 weeks post-treatment for evaluable population, % (n/total)		
Rate, % (n/total)	79 (112/142)	31 (23/75)
Age		
≥ 65 years	75 (63/84)	20 (9/45)
< 65 years	85 (49/58)	47 (14/30)
Sex		
Female	82 (72/88)	33 (16 /48)
Male	74 (40/54)	26 (7/27)
Number of prior CDI episodes^1^		
≤ 3 CDI episodes	75 (54/72)	33 (22/67)
> 3 CDI episodes	83 (58/70)	20 (1/5)
Laboratory test for enrollment^1^		
PCR	77 (73/95)	31 (19/61)
EIA	81 (26/32)	29 (2/7)
Other^2^	87 (13/15)	50 (2/4)
Antibiotic at enrolling CDI episode^1^		
Vancomycin	79 (92/117)	29 (13/45)
Fidaxomicin	50 (3/6)	25 (1/4)
Metronidazole	100 (8/8)	20 (1/5)
Other^3^	82 (9/11)	50 (9/18)
Sustained CDI recurrence-free rates, % (n/total)		
6 months post-treatment	97 (109/112)	NC
12 months post-treatment	95 (101/106)	NC
24 months post-treatment	91 (88/97)	NC

^1^Five historical control participants in the FAS (3 participants in evaluable population) had incomplete CDI history records and were not able to be categorized

^2^Category ‘other’ for diagnostics included medical record documentation as antigen, empirically tested, loop-mediated isothermal amplification (LAMP), molecular assay, nucleic acid amplification, toxin, toxin with PCR reflex, not reported, or unknown

^3^Category ‘other’ for Antibiotics included medical record documentation as any combination of vancomycin, fidaxomicin, metronidazole, rifampin, and rifaximin, as well as unknown and not reported

Among the RBX2660-treated evaluable population, efficacy was not significantly different (*p* > 0.05, Fisher’s exact test) amongst demographic subgroups of age (≥ 65 vs < 65), sex, number of CDI episodes (≤ 3 episodes versus > 3 episodes), laboratory test for enrollment, or antibiotic for enrolling CDI episode (vancomycin, fidaxomicin, other). Among the RBX2660-treated Evaluable Population—97% of 112 evaluable primary responders remained CDI-free at 6 months after treatment, with 95% and 91% of evaluable responders remaining CDI-free at 12 and 24 months, respectively, after the last received treatment.

### Safety

Of the 149 participants treated with RBX2660, 123 (83%) experienced a total of 805 treatment-emergent AEs (TEAEs). The majority of TEAEs were mild to moderate in severity (Table 3). The frequency of reported AEs was similar among age and sex groups. TEAEs were primarily related to pre-existing condition(s), with 58% of participants experiencing such events. Next most common were TEAEs related to *C. difficile* disease, experienced by 35% of participants. TEAEs related to RBX2660 were experienced by 21% of participants. Temporally, 60% of participants experienced TEAEs in the RBX2660-treated group during the first 4 weeks after treatment.

**Table 3:** Treatment emergent adverse events (AE) among Safety Population (n = 149)

TEAE, events/participants (% of participants)	
Overall	805/123 (83)
Onset interval	
Baseline to 4 weeks	249/89 (60)
4–8 weeks	78/40 (27)
8 weeks to 3 months	55/36 (24)
3–6 months	133/53 (36)
6–12 months	141/59 (40)
12–24 months	147/45 (30)
Age	
< 65 years old	322/50 (81)
≥ 65 years old	483/73 (84)
Race	
White	720/113 (83)
Non-white	85/10 (77)
Sex	
Female	472/81 (85)
Male	333/42 (78)
Severity	
Mild	435/31 (21)
Moderate	242/49 (33)
Severe	100/36 (24)
Potentially life threatening	28/19 (13)
Relatedness	
Related to investigational product^1^	67/32 (22)
Related to enema procedure^1^	44/24 (16)
Related to *C. difficile* disease^1^	137/52 (35)
Related to pre-existing condition^1^	388/87 (58)
SAE, events/participants (% of participants)	
Total	208/52 (35)
Related to investigational product	9/2 (1)
Related to enema procedure	4/1 (1)
Related to *C. difficil*e disease	31/16 (11)
Related to pre-existing condition	123/38 (26)
Leading to death	20/15 (10)
Related to investigational product or procedure^2^	1/1 (1)
Related to pre-existing condition	12/11 (7)

^1^Defined as possibly, probably, or definitely related

^2^One death (SAE) possibly related to investigational product and enema procedure, definitely related to *C. difficil*e disease and pre-existing condition

The most common AE system organ class was gastrointestinal disorders, with diarrhea reported in 30% of participants (Table 4). Most of these occurred during the first 8 weeks after first RBX2660 dose, coincident with CDI recurrences among treatment non-responders. There were also 20 participants who reported urinary tract infections (UTIs), of which eight had a documented medical history of at least one UTI. Nineteen of the 20 participants were treated with antibiotics for their UTI. None of the UTI TEAEs were deemed related to RBX2660 or the enema procedure. There were no reported gastrointestinal infections for which the causative pathogen was traced to RBX2660.

**Table 4:** Adverse events occurring in ≥ 5% of participants classified by system organ class and preferred term

Events/participants (% of participants)			
System organ class and preferred term	Treatment through study exit	Baseline to 4 weeks	4 to 8 weeks
Gastrointestinal disorders			
Diarrhea	72/44 (30)	36/29 (20)	10/10 (7)
Abdominal pain	32/18 (12)	25/17 (11)	0 (0)
Constipation	22/17 (11)	15/12 (8)	1/1 (1)
Nausea	15/10 (7)	7/5 (3)	0 (0)
Abdominal distension	11/9 (6)	11/9 (6)	0 (0)
Flatulence	9/8 (5)	9/8 (5)	0 (0)
General disorders and administration site conditions			
Pyrexia	11/9 (6)	8/7 (5)	0 (0)
Infections and infestations			
Urinary tract infection	26/20 (13)	4/4 (3)	4/4 (3)
Pneumonia	10/10 (7)	0 (0)	0 (0)
Sepsis	10/8 (5)	0 (0)	1/1 (1)
Upper respiratory tract infection	9/8 (5)	3/3 (2)	2/2 (1)
Nervous system disorders			
Headache	9/9 (6)	7/7 (5)	0 (0)
Psychiatric disorders			
Anxiety	8/8 (5)	3/3 (2)	0 (0)

Throughout the 24-month follow-up period, there were 208 treatment-emergent SAEs in 35 participants. Of these, nine events were assessed as possibly related to RBX2660, from two participants, including ileus, leukocytosis, pyrexia, atrial fibrillation, coincident with severe CDI eventually leading to death in one participant and three CDI episodes (one severe) in another participant. All nine SAEs from these two participants were possibly or definitely related to *C. difficile* disease and/or pre-existing condition(s). There were 15 deaths among the treated population in the 24 months of follow-up with a median time to onset of TEAEs leading to death was 407 days after completing study treatment. Three participants had deaths which occurred within 6 months of completing RBX2660 treatment. These 3 participants were all over 65 years of age and had extensive medical histories, including cardiopulmonary diseases. One of these participants had a TEAE leading to death (a severe CDI recurrence) which was attributed as possibly related to RBX2660 and definitely related to both pre-existing conditions and CDI. 

### Microbiome analysis of RBX2660-treated participants

Of 142 participants in the Evaluable Population, 127 provided at least one fecal sample that was included in this analysis, including 105 responders and 22 non-responders and spanning from before RBX2660 treatment (baseline) to 24 months after treatment. In addition, 155 RBX2660 samples were included, representing all administered doses.

**Fig. 2:**
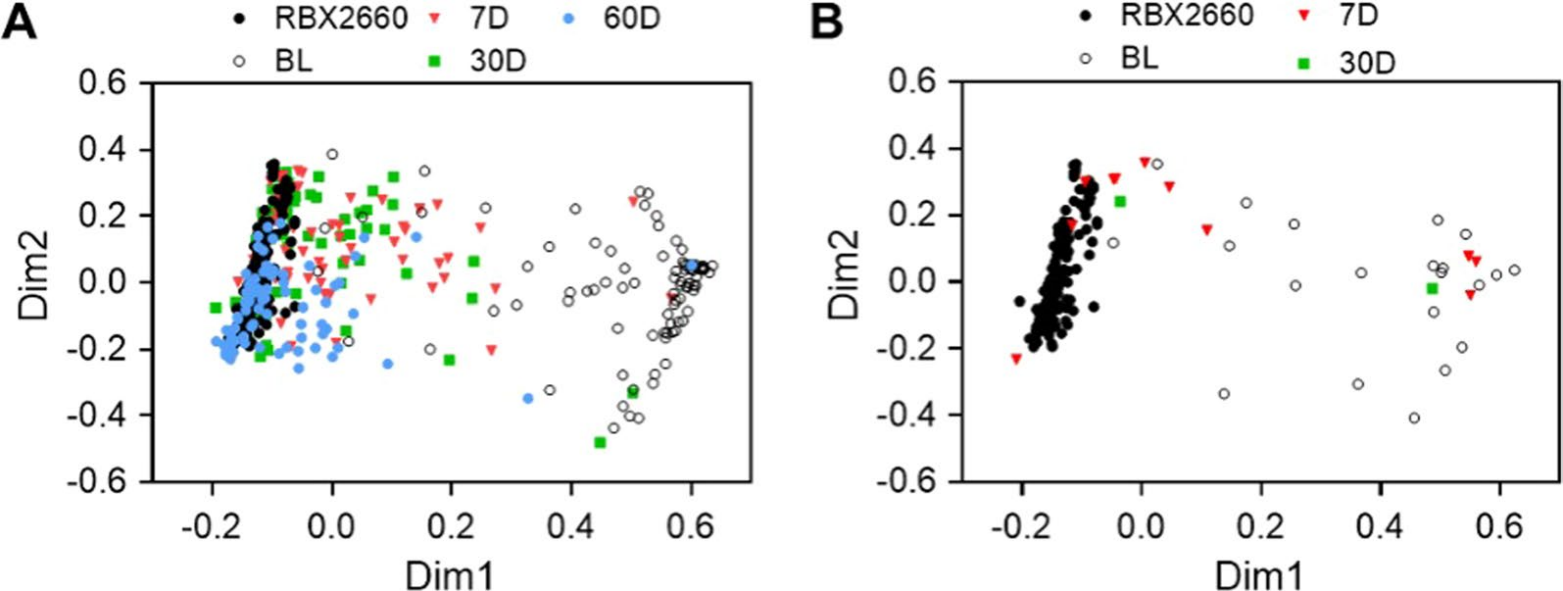
Non-parametric multidimensional similarity analysis (NDMS) based on Bray–Curtis dissimilarity for microbiome compositions of RBX2660 and responder microbiome compositions before treatment (BL) and 1, 4, and 8 weeks after last received RBX2660 treatment. **A** Treatment responders. **B** Treatment non-responders

Non-parametric multidimensional scaling analysis (NMDS) indicated that responders’ overall microbiome compositions were highly divergent from the RBX2660 composition prior to treatment (‘BL’, Fig. 2) and converged toward the RBX2660 composition within 7 days after treatment (7D), with increasing convergence observed up to 60 days after treatment. The similarity of participants microbiomes to RBX2660 was durable to at least 24 months after treatment (Additional file 1: Fig. S2).

**Fig. 3:**
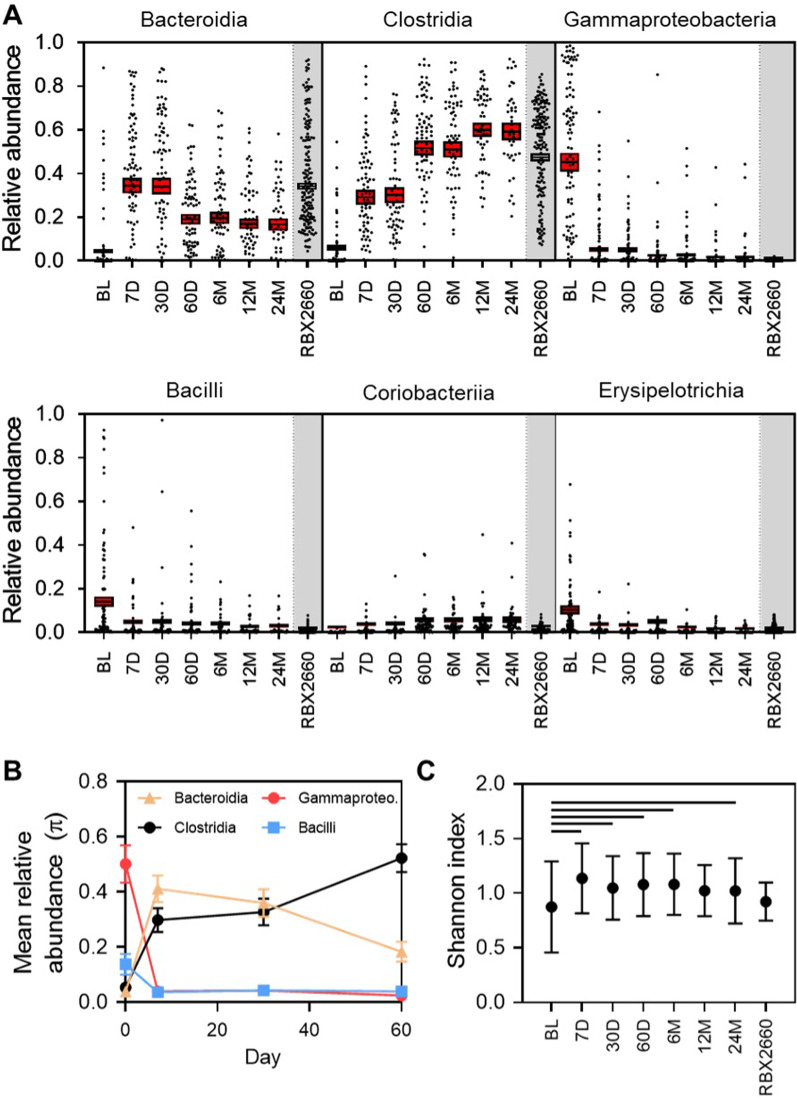
Microbiome composition and alpha diversity for RBX2660 and responders before and after treatment. **A** Sample and group mean (π) relative abundances at the class level for classes comprising at least 3% relative abundance at one or more time points, denoted as before treatment (BL), 7, 30, 60 days after treatment (7D, 30D, 60D), or 6, 12, 24 months after last received RBX2660 treatment (6 M, 12 M, 24 M). Individual samples are represented as dots, and group means (π) with upper and lower confidence intervals (red boxes) were calculated based on maximum likelihood estimate using the Dirichlet multinomial. **B** Group mean relative abundances (π) with upper and lower confidence intervals for subset of participants who provided all four time points shown—a repeated measurements analysis. **C** Alpha diversity of RBX2660 and participant samples for each time point group, expressed as the mean and standard deviation of the Shannon indices. Lines with an asterisk (*) indicate statistically significant differences (*p* < 0.05, *t*-test) between two time point groups

At the taxonomic class level, Gammaproteobacteria, Bacilli, and Erysipelotrichia predominated before treatment, with depleted Bacteroidia and Clostridia relative to RBX2660 (Fig. 3A). Among responders, Bacteroidia and Clostridia were restored to predominance, and Gammaproteobacteria, Bacilli, and Erysipelotrichia decreased to become more similar to the composition of RBX2660 within 7 days after treatment, and these changes were sustained to at least 24 months after treatment. Although there was convergence toward RBX2660, participants’ microbiomes remained distinct from RBX2660 at all time points (*p* < 0.001, permutation test). A repeated measurements subgroup analysis—a microbiome pharmacodynamic measure [8, 9]—was conducted for the subset of 35 participants who provided all four sample time points from BL, 7, 30, and 60 days after treatment. This average within-participant analysis confirmed the directionality, rate, and consistency of these taxonomic changes (Fig. 3B). Concurrent with taxonomic changes, alpha diversity at the class level increased significantly among responders from before to 7 days after treatment (*p* < 0.05, paired *t*-test; Fig. 3C) and remained significantly higher (*p* < 0.05) at all post-treatment time points except 12 months (*p* = 0.127).

**Fig. 4:**
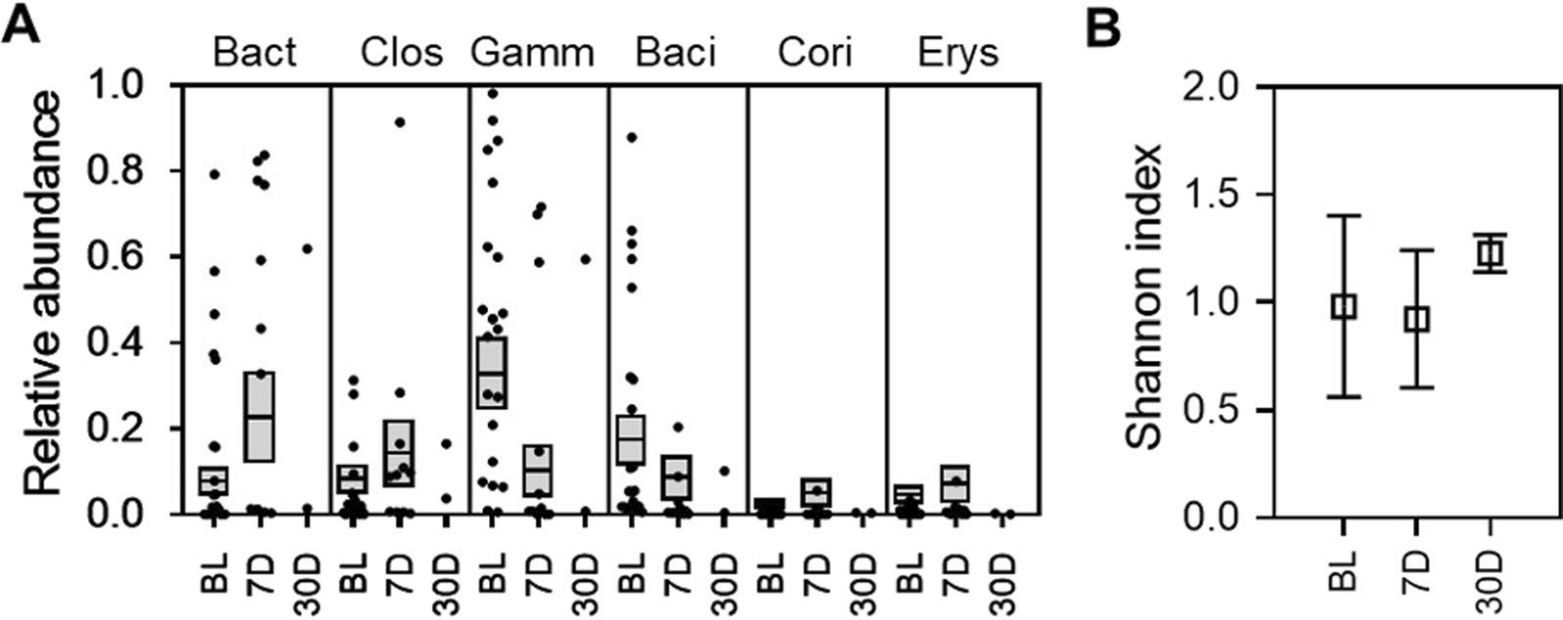
Microbiome composition and alpha diversity for non-responders before and after treatment. **A** Sample and group mean (π) relative abundances at the class level for classes comprising at least 3% relative abundance at one or more time points, denoted as before treatment (BL) and 7 and 30 days after last received RBX2660 treatment (7D, 30D). Class abbreviations refer to the taxonomic classes in Fig. 3. **B** Alpha diversity of non-responders for each time point group, expressed as the mean and standard deviation of the Shannon indices. There were no significant differences among the time points

Non-responders’ microbiome compositions were not significantly different from responders’ at baseline (*p* = 0.429, permutation test, and Additional file 1: Fig. S3) but were highly divergent from RBX2660 (Fig. 2B). At seven and 30 days after treatment non-responders’ microbiomes showed little convergence with RBX2660, noting that sample numbers were limited. At the taxonomic class level, non-responders showed less reduction of Gammaproteobacteria and Bacilli and restoration of Clostridia and Bacteroidia after treatment (Fig. 4A). Likewise, there was no significant change in alpha diversity from before to seven or 30 days after treatment among non-responders (*p* > 0.05, *t*-test; Fig. 4B). As noted above, sample size in the non-responder time groups were small (n = 11 at 7 days and n = 2 at 30 days).

**Fig. 5:**
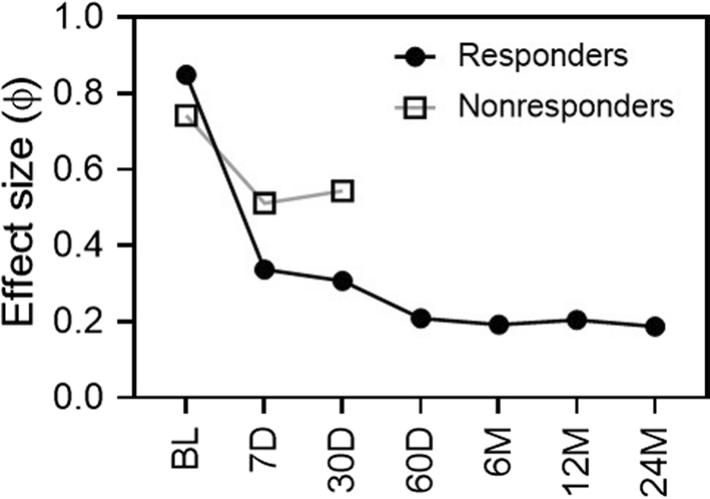
Effect size of each time point group of responders and non-responders compared with RBX2660. Effect size is expressed as a modified Cramer criterion, ϕ, for each pairwise comparison

As a broad-consortium microbiota suspension sourced from healthy human donors with minimal processing, RBX2660 can be considered representative of a healthy microbiome. Therefore, as an estimate of the effectiveness of RBX2660 to restore participants’ microbiota, the similarity of participants’ microbiomes to RBX2660 was quantified at all time points using a modified Cramer’s effect size criterion (ϕ, [6, 7]). Effect size expresses the (dis)similarity between two microbiome populations, with an effect size of zero indicating identical populations and an effect size of < 0.2 indicating a small difference [10]. Prior to treatment, effect size of responders and non-responders compared to RBX2660 was high (Fig. 5). After treatment effect size decreased more for responders than non-responders, reaching a small effect size by 60 days that was durable to at least 24 months.

## Discussion

RBX2660 is an investigational microbiota-based live biotherapeutic that has been evaluated in two previous clinical trials for reducing rCDI recurrence, including an open-label [6] and a placebo-controlled study for which an interim analysis has been published [11], as well as two Phase 3 trials for which results have not yet been published (NCT03931941, NCT03244644). Here we report the final study data, demonstrating a treatment success rate of 78.9% with RBX2660 compared to 30.7% treatment success in the historical control group. This RBX2660 treatment success rate compares favorably to the prior open-label analyses of RBX2660 [6, 11] and to the 83% aggregated rate for FMT in prospective open-label studies [12]. The reported RBX2660 success rate is also comparable to a real-world FMT practice, as estimated in an observational study by the American Gastroenterological Association registry [13]. Importantly, the recurrence-free rate after RBX2660 is better than after antibiotic treatment alone, measured as 30.7% in the matched historical control group of this study and estimated as 40–60% in the literature [14, 15]. For the historical control, it should be noted that treatment outcomes were only assigned for participants for whom recurrence or recurrence-free status was unequivocally documented, which resulted in exclusion of 28% of the full analysis set. This correction was not applied to an interim data cut of this study, which has been previously reported [16]. Additionally, because the participants were not randomized to active or control, there is a possibility of a selection bias between the two groups. The impact of this conservative approach on the recurrence-free rate cannot be ascertained. Finally, to our knowledge this is the first trial to report post-treatment CDI-free rates over a 24-month period, with 91% of evaluable primary responders remaining CDI occurrence-free to 24 months. If confirmed in future studies, this is an important precedent that microbiota-directed approaches can provide long term benefit for preventing rCDI.

Our study also adds to the body of safety data for RBX2660, including an extended follow-up of 24 months after treatment. Overall, 84% of TEAEs were mild to moderate severity, which is consistent with the two prior trials of RBX2660 [6, 11]. Only two participants reported SAEs assessed as possibly related to RBX2660, and these SAEs were also deemed related to pre-existing conditions and CDI. Deaths during study occurred in 15 of 149 patients (10.1%), and the median time to onset of the TEAE leading to death was 407 days after completing study treatment. Epidemiological estimates of 30-day in-hospital, all-cause mortality rates for CDI patients range from 1.3 to 9.3% (depending on if the infection was healthcare-assocaited or not) [18]. One participant had a TEAE leading to death (a severe CDI recurrence) which was attributed as possibly related to RBX2660 and definitely related to both pre-existing conditions and CDI. This participant was of advanced age, with cardiovascular comorbidities, and experienced a recurrence of CDI, all of which are unfortunately known contributors to risk of death in CDI patients [17]. During manufacturing, RBX2660 is rigoursly screened for known pathogens, including *C. difficile*, to ensure no transmission of pathogens. The event was reviewed by the independent Medical Monitor and determined to not be a product-related safety concern.

The safety profile in this trial is also consistent with observational studies of FMT, which report post-administration symptoms as being generally mild, recognizing that most studies did not require prospective or standardized safety monitoring [13, 16]. In general, most serious safety events associated with FMT have been related to colonoscopic administration procedures [19]. There are also documented cases of serious infectious pathogens directly transmitted via FMT, including bacteremia caused by extended-spectrum β-lactamase-(ESBL)-producing *Escherichia coli* that resulted in death [20] and Shiga toxin-producing *E. coli* (STEC) [21]. In those cases, the FMT product was not screened for the infecting organism, underscoring the value of mandated and continuous evolution of pathogen screening practices. In the present trial, there were no reported infections caused by pathogens traceable to RBX2660, consistent with prior studies.

In this study, clinical reduction of CDI recurrence by RBX2660 also correlated with restorative microbiome changes—significantly increased abundance of Bacteroidia and Clostridia-class bacteria, decreased abundance of Gammaproteobacteria and Bacilli, and increased alpha diversity. These changes are consistent with previously reported results for RBX2660 [3] and RBX7455—a lyophilized non-frozen microbiota formulation [8]. Similar microbiome changes have been observed after FMT [22, 23]. In this study, these changes resulted in rapidly increased similarity to the administered RBX2660, expressed as a decreased effect size, and this similarity was sustained to the end of the 24-month post-treatment follow-up. It is noted that this method did not aim to measure strain-level engraftment from RBX2660. Thus, the resulting healthier composition in participants may have arisen from strains present in RBX2660, in the patient, or a mixture. For reducing rCDI, durable strain-level engraftment is probably less important than simply the instillation and presence of healthy microbiota and its metabolic activities during a time window of vulnerability to recurrence [3]. The subset of participants with full longitudinal microbiome data to 60 days – a pharmacodynamic analysis via repeated measures indicates that the taxonomic changes occurred mostly during the first 7 days.

The principal limitation of this study, acknowledged during the design stage, were the open-label design and exclusion of patients with certain comorbidities common to the rCDI population—inflammatory bowel disease and irritable bowel syndrome. Subsequent trials have been initiated that will provide more data from a randomized, double-blinded, placebo-controlled design (NCT03244644) as well as with patients with these selected comorbidities (NCT03931941). Despite these limitations, the results herein provide further support for the safety, efficacy, favorable microbiome outcomes, and potential benefit of RBX2660 to reduce the healthcare burden related to rCDI.

## Supplementary Information

**Additional file 1.** **Table S1.** Number of samples included in microbiome analysis by time point. **Figure S1.** Kaplan-Meier analysis plot of recurrence-free participants over time after last received RBX2660 treatment. igure S2. Non-parametric multidimensional similarity analysis (NMDS) based on Bray-Curtis dissimilarity for microbiome compositions of RBX2660 and responder microbiome compositions before treatment (BL) and 6, 12, and 24 months (top, middle, and bottom panels, respectively) after last received RBX2660 treatment. **Figure S3.** Non-parametric multidimensional similarity analysis (NMDS) based on Bray-Curtis dissimilarity for microbiome compositions of responder and nonresponder microbiome compositions before treatment (BL). There was no signficant difference (p > .05, parametric t-test).

## Data Availability

The datasets generated and/or analyzed during the current study are not publicly available due to pending regulatory submission and approval but are available from RO on reasonable request.

## Abbreviations

CDI: *Clostridioides difficile* Infection
rCDI: Recurrent *Clostridioides difficile* infection

## References

[1] Guh AY, Mu Y, Winston LG, Johnston H, Olson D, Farley MM (2020). Trends in U.S. Burden of *Clostridioides difficile* infection and outcomes. N Engl J Med.

[2] Johnson S, Lavergne V, Skinner AM, Gonzales-Luna AJ, Garey KW, Kelly CP, Wilcox MH (2021). Clinical Practice Guideline by the Infectious Diseases Society of America (IDSA) and Society for Healthcare Epidemiology of America (SHEA): 2021 Focused Update Guidelines on Management of *Clostridioides difficile* Infection in Adults. Clin Infect Dis.

[3] Blount KF, Shannon WD, Deych E, Jones C (2019). Restoration of bacterial microbiome composition and diversity among treatment responders in a phase 2 trial of RBX2660: an investigational microbiome restoration therapeutic. Open Forum Infect Dis.

[4] Bafeta A, Yavchitz A, Riveros C, Batista R, Ravaud P (2017). Methods and reporting studies assessing fecal microbiota transplantation: a systematic review. Ann Intern Med.

[5] Khanna S, Pardi DS, Kelly CR, Kraft CS, Dhere T, Henn MR (2016). A novel microbiome therapeutic increases gut microbial diversity and prevents recurrent *Clostridium difficile* infection. J Infect Dis.

[6] Orenstein R, Dubberke E, Hardi R, Ray A, Mullane K, Pardi DS (2016). Safety and durability of RBX2660 (Microbiota Suspension) for recurrent *Clostridium difficile* infection: results of the PUNCH CD study. Clin Infect Dis.

[7] La Rosa PS, Brooks JP, Deych E, Boone EL, Edwards DJ, Wang Q (2012). Hypothesis testing and power calculations for taxonomic-based human microbiome data. PLoS ONE.

[8] Khanna S, Pardi DS, Jones C, Shannon WD, Gonzalez C, Blount KF (2020). RBX7455, a non-frozen, orally-administered investigational live biotherapeutic, is safe, effective, and shifts patients’ microbiomes in a phase 1 study for recurrent *Clostridioides difficile* infections. Clin Infect Dis.

[9] Shannon WD. Repeated Measures Method for Microbial Count Data (BioRankings Technical Report #3).2017.

[10] Cohen J (1992). A power primer. Psychol Bull.

[11] Dubberke ER, Lee CH, Orenstein R, Khanna S, Hecht G, Gerding DN (2018). Results from a randomized, placebo-controlled clinical trial of a RBX2660-A microbiota-based drug for the prevention of recurrent *Clostridium difficile* infection. Clin Infect Dis.

[12] Tariq R, Pardi DS, Bartlett MG, Khanna S (2019). Low cure rates in controlled trials of fecal microbiota transplantation for recurrent clostridium difficile infection: a systematic review and meta-analysis. Clin Infect Dis.

[13] Kelly CR, Yen EF, Grinspan AM, Kahn SA, Atreja A, Lewis JD (2020). Fecal microbiota transplant is highly effective in real-world practice: initial results from the FMT national registry. Gastroenterology.

[14] Kelly CP (2012). Can we identify patients at high risk of recurrent *Clostridium difficile* infection?. Clin Microbiol Infect.

[15] Sheitoyan-Pesant C, Abou Chakra CN, Pepin J, Marcil-Heguy A, Nault V, Valiquette L (2016). Clinical and healthcare burden of multiple recurrences of *Clostridium difficile* infection. Clin Infect Dis.

[16] Orenstein R, Dubberke ER, Khanna S, Hecht G, Dupont H, Lee C (2017). RBX2660 is safe, superior to antibiotic-treated controls for preventing recurrent *Clostridium difficile*, and may rehabilitate patient microbiomes: open label trial results. Open Forum Inf Dis..

[17] Olsen MA, Yan Y, Reske KA, Zilberberg MD, Dubberke ER (2015). Recurrent *Clostridium difficile* infection is associated with increased mortality. Clin Microbiol Infect.

[18] Lessa FC, Winston LG, McDonald LC (2015). Emerging infections program *C. difficile* surveillance team. Burden of Clostridium difficile infection in the United States. N Engl J Med.

[19] Wilcox MH, McGovern BH, Hecht GA (2020). The efficacy and safety of fecal microbiota transplant for recurrent clostridium difficile infection: current understanding and gap analysis. Open Forum Infect Dis.

[20] Drekonja D, Reich J, Gezahegn S, Greer N, Shaukat A, MacDonald R (2015). Fecal microbiota transplantation for clostridium difficile infection: a systematic review. Ann Intern Med.

[21] DeFilipp Z, Bloom PP, Torres Soto M, Mansour MK, Sater MRA, Huntley MH (2019). Drug-resistant *E. coli* bacteremia transmitted by fecal microbiota transplant. N Engl J Med.

[22] Zellmer C, Sater MRA, Huntley MH, Osman M, Olesen SW, Ramakrishna B (2021). Shiga TOxin producing *Escherichia coli* transmission via fecal microbiota transplant. Clin Infect Dis.

[23] Staley C, Kelly CR, Brandt LJ, Khoruts A, Sadowsky MJ. Complete microbiota engraftment is not essential for recovery from recurrent clostridium difficile infection following fecal microbiota transplantation. mBio. 2016;7(6). 10.1128/mBio.01965-16 PMC5181777 27999162

[24] Seekatz AM, Aas J, Gessert CE, Rubin TA, Saman DM, Bakken JS (2014). Recovery of the gut microbiome following fecal microbiota transplantation. MBio.

