# Supplementary material for: Durable reduction of Clostridioides difficile infection recurrence and microbiome restoration after treatment with RBX2660: results from an open-label phase 2 clinical trial
Source: BMC Infect Dis. 2022 Mar 12;22:245. doi: 10.1186/s12879-022-07256-y (PMC8917640; doi:10.1186/s12879-022-07256-y)
Supplement: Supplementary file 1 — Additional file 1. Table S1. Number of samples included in microbiome analysis by time point. Figure S1. Kaplan-Meier analysis plot of recurrence-free participants over time after last received RBX2660 treatment. igure S2. Non-parametric multidimensional similarity analysis (NMDS) based on Bray-Curtis dissimilarity for microbiome compositions of RBX2660 and responder microbiome compositions before treatment (BL) and 6, 12, and 24 months (top, middle, and bottom panels, respectively) after last received RBX2660 treatment. Figure S3. Non-parametric multidimensional similarity analysis (NMDS) based on Bray-Curtis dissimilarity for microbiome compositions of responder and nonresponder microbiome compositions before treatment (BL). There was no signficant difference (p > .05, parametric t-test). [file 12879_2022_7256_MOESM1_ESM.docx]

**Table S1**. Number of samples included in microbiome analysis by time point.

|  | BL | 7D | 30D | 60D | 6M | 12M | 24M | RBX2660 |
| --- | --- | --- | --- | --- | --- | --- | --- | --- |
| Responders | 90 | 74 | 67 | 75 | 63 | 54 | 41 | 155 |
| Non-responders | 21 | 11 | 2 |  |  |  |  |  |


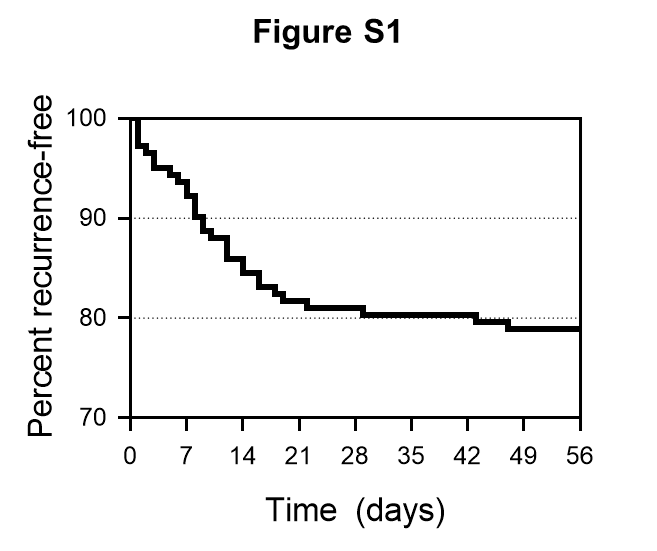


**Figure S1**. Kaplan-Meier analysis plot of recurrence-free participants over time after last received RBX2660 treatment.


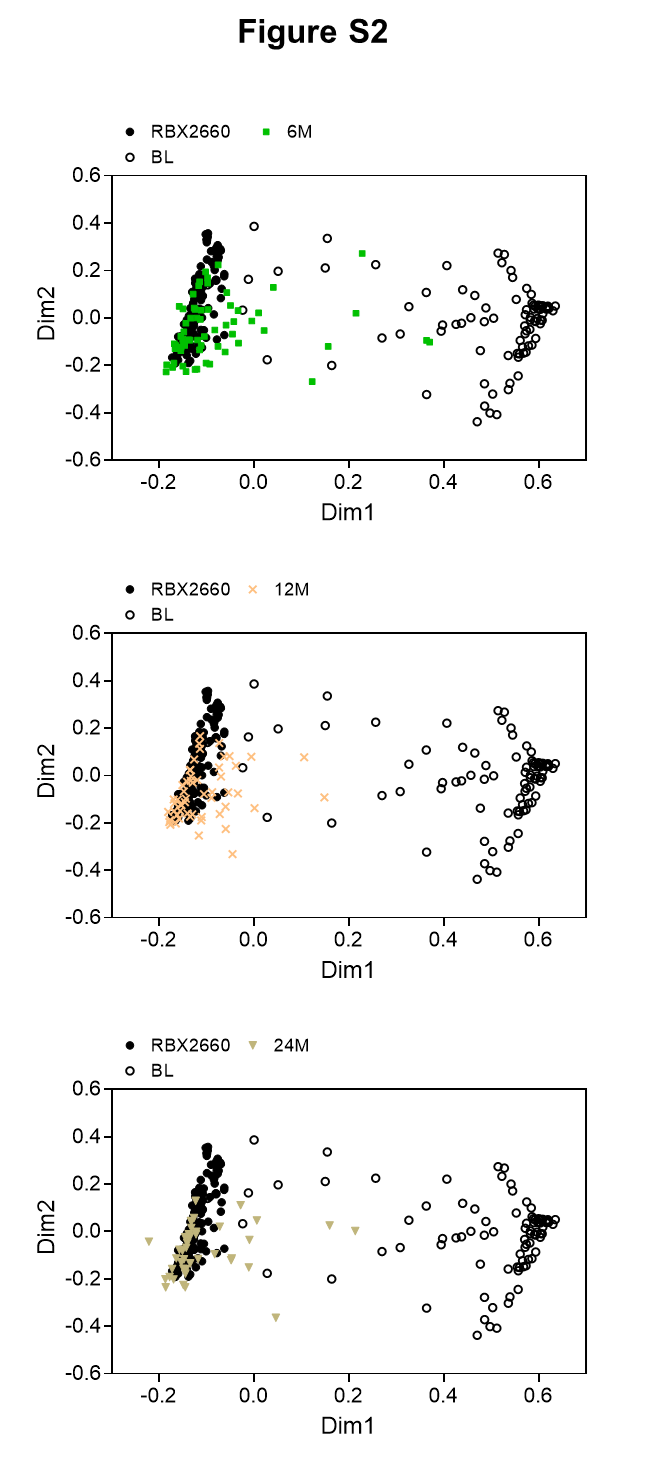


**Figure S2**. Non-parametric multidimensional similarity analysis (NMDS) based on Bray-Curtis dissimilarity for microbiome compositions of RBX2660 and responder microbiome compositions before treatment (BL) and 6, 12, and 24 months (top, middle, and bottom panels, respectively) after last received RBX2660 treatment.


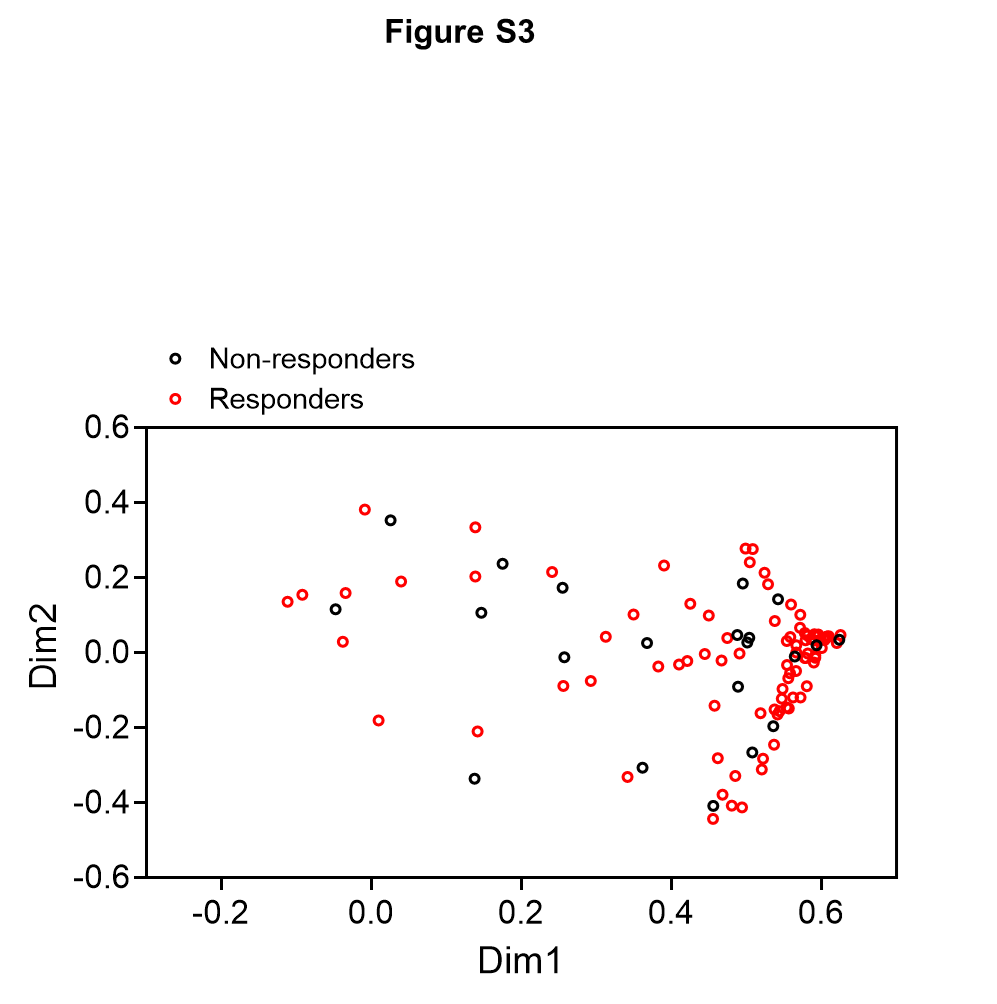


**Figure S3**. Non-parametric multidimensional similarity analysis (NMDS) based on Bray-Curtis dissimilarity for microbiome compositions of responder and nonresponder microbiome compositions before treatment (BL). There was no signficant difference (*p* > .05, parametric t-test).
